# Transcriptome Analysis Reveals the Involvement of *PnMYB26* in Regulating Anther Development in *Phyllostachys nigra*

**DOI:** 10.3390/biology15131049

**Published:** 2026-07-01

**Authors:** Feiyi Huang, Yujie Mao, Jiaxin Wang, Chao Tang

**Affiliations:** 1 State Key Laboratory for Development and Utilization of Forest Food Resources, Nanjing Forestry University, Nanjing 210037, China; 2 Co-Innovation Center for Sustainable Forestry in Southern China, Nanjing Forestry University, Nanjing 210037, China; 3 Bamboo Research Institute, Nanjing Forestry University, Nanjing 210037, China; 4 Sanya Institute of Nanjing Agricultural University, National Key Laboratory of Crop Genetics & Germplasm Enhancement and Utilization, Nanjing Agricultural University, Nanjing 210095, China

**Keywords:** *Phyllostachys nigra*, anther, development, MYB26, transcriptome

## Abstract

*Phyllostachys nigra* is an important ornamental plant characterized by deep purple culms and a long flowering period. Normal anther development is an essential prerequisite for seed formation in plants. The regulatory mechanism of the development of anther in *P. nigra* is still not clear. To better understand the anther development process in *P. nigra*, we compared the transcriptome analysis of anthers at four different developmental stages. We selected 1528 differentially expressed genes (DEGs) during these four stages. KEGG and WGCNA analyses revealed that the DEGs were mainly involved in ‘cutin, suberine and wax biosynthesis’ and ‘phenylpropanoid biosynthesis’. Moreover, 125 transcription factors were differentially expressed across these four stages, among which the MYB family harbored the largest number of members. We performed qPCR to validate the selected candidate genes. The overexpression of *PnMYB26* may modulate *NST1* expression, a gene associated with secondary cell wall thickening, thereby inhibiting pollen release and ultimately reducing fertility in *Arabidopsis*. Our results uncovered the regulatory mechanism of anther development and helped us to further explore the regulatory mechanisms of anther development-related genes in *P. nigra*.

## Simple Summary

*Phyllostachys nigra* is an ornamental bamboo characterized by a long flowering cycle, while the molecular mechanism of its anther development remains elusive. We profiled gene expression in anthers across four developmental stages and identified 1528 genes associated with anther development. These genes participate in the biosynthesis of cutin, wax and phenylpropanoids, which are essential for pollen development and anther dehiscence. The heterologous overexpression of *PnMYB26* in *Arabidopsis* affects pollen release and reduces fertility. This study may establish a transcriptomic resource for anther development in *P. nigra* and offer promising candidate genes for subsequent breeding programs.

## 1. Introduction

The anther is indispensable for reproductive success via the formation and release of pollen grains. In rice, anther development comprises a series of sequential events that is tightly regulated with an elaborate regulatory network [1]. In the early stage, stamen primordium cells proliferate and then differentiate into pollen mother cells (PMCs). The PMCs are surrounded by the epidermis, endothecium, and middle and tapetum layers [2]. The endothecium forms lignin-deposited secondary thickening to promote anther dehiscence [3]. During meiosis, PMCs generate four haploid microspores, which are initially encapsulated in a callose wall to form tetrads [4]. The tapetum synthesizes callase to degrade the callose wall, thereby releasing single microspores into the anther locule [5]. As development proceeds, microspores experience cell expansion, vacuolation and two mitotic cycles. Mature pollen grains are ultimately formed, with two sperm cells embedded in a nutrient-abundant vegetative cell, and the peripheral pollen wall thickens steadily until full pollen maturity [6]. Alongside microspore development after meiosis, programmed cell death occurs in the tapetum, generating and secreting precursors essential for pollen maturation and pollen wall formation [7].

Many evolutionarily conserved genes related to anther development have been reported in *Arabidopsis* and rice [6]. *FAR2* and *CYP704B1* have been reported to regulate cuticle and exine formation in anther [8,9]. As an important metabolic pathway, phenylpropanoid metabolism yields various secondary metabolites related to anther development, like lignin and flavonoids [10]. Many genes in this pathway play pivotal regulatory roles in modulating anther development, like *Gh4CL20* [11], *CCR1*, and *CAD* genes [12]. Numerous genes play roles in transport processes are also essential for pollen fertility, such as *AtABCG26* [13] and *OsABCG26* [14]. Transcription factors are core regulators of gene expression networks. They bind to specific *cis*-elements in target gene promoters to modulate plant development and stress responses. As key hubs integrating endogenous developmental signals and external environmental stimuli, transcription factors can activate or repress individual genes and remodel regulatory networks, facilitating plant adaptation [15]. Thus, the identification and functional characterization of pivotal transcription factors are critical to unraveling the molecular mechanisms underlying anther maturation and dehiscence. Among the largest transcription factor families in plants, MYBs are involved in many developmental processes, like anther and trichome development [16]. In anther development, several *MYB*s (like *MYB103* and *MYB26*) regulate tapetum differentiation or endothecial secondary wall thickening, but their regulatory roles in non-model plants remain poorly understood. In *Arabidopsis*, *Tapetal Development and Function 1* (*TDF1*/*MYB35*) and *Male Sterility 188* (*MS188*/*MYB103*) precisely control tapetal development [4]. The mutation of *AtMYB103* causes the abnormal development of exine and pollen coat in microspores, ultimately triggering microspore abortion [17]. *OsMYB103*, the ortholog of *AtMYB103*, shares similar expression profiles and functional roles in rice [18]. In addition to these *MYB* genes, *MYB33*, *MYB65* and *MYB26* also play key roles in anther development. *atmyb33* and *atmyb65* mutants exhibit male sterility, which is caused by impaired tapetum degeneration [19]. During anther development, the endothecium undergoes secondary wall thickening (visualized by phloroglucinol-HCl or toluidine blue), which generates mechanical force for dehiscence upon desiccation. In *Arabidopsis*, MYB26 regulates anther secondary wall thickening by directly promoting *NST*s [20]. Both the overexpression and loss of function of *MYB26* cause abnormal anther dehiscence [21]. However, it still remains unclear whether *MYB*s contribute to anther development in *Phyllostachys nigra*.

The transcriptomic analysis of gene expression reveals the molecular mechanisms underlying anther development. In *Arabidopsis*, 1587 DEGs are identified in mature pollen based on *Arabidopsis* microarray data [22]. The transcriptome of anther development has also been investigated in rice [23]. The study characterized a ‘U-shaped’ trend in rice stage-specific transcripts, of which the fewest genes show preferential expression at the bicellular pollen stage. In rice, clear transcriptome divergence between the male gametophyte and tapetum has been revealed through laser-microdissected cell sampling [24]. Large-scale transcriptome analysis has clearly revealed gene expression patterns during anther development in *Arabidopsis* and rice. However, the genes involved in bamboo anther development remains poorly understood, especially in *P. nigra*. As a member of Poaceae, *P. nigra* is a non-timber forest product of great cultural and economic significance. *P. nigra* has a long flowering cycle and a low seed-setting rate, which severely restricts its breeding process [25].

In this study, we adopted the anther developmental classification criteria established in *Arabidopsis* and rice [6]. The four developmental stages of *P. nigra* anthers were confirmed through histological section analysis, and subsequent transcriptome sequencing was carried out for further research. A total of 1528 DEGs were identified. KEGG pathway enrichment was performed to characterize the pivotal metabolic pathways during anther development. Integrating expression trend analysis, WGCNA, differentially expressed transcription factor screening and homologous gene functional annotation, we prioritized *PnMYB26* within the MYB family. *PnMYB26*, a homolog of *AtMYB26*, was cloned for subsequent functional characterization. The overexpression of *PnMYB26* caused a significant decline in fertility. These results provide novel insights into the regulatory mechanisms and genetic basis of anther development in bamboo.

## 2. Materials and Methods

### 2.1. Plant Material

During flowering, the anthers were collected from the Dayu Scenic Bamboo Garden, Yangzhou, Jiangsu, China (119°38′ E, 32°29′ N) for paraffin embedding and sectioning. The anthers were harvested in four stages: microspore mother cell (S1), tetrad (S2), uninucleate microspore (S3), and binucleate pollen stage (S4) based on paraffin section microscopic observations. The anthers of each stage had three biological replicates. Samples were rapidly frozen and stored at −80 °C. *Arabidopsis* (col-0) and tobacco (*Nicotiana benthamiana*) were placed in the chamber under a photoperiod of 16 h day/8 h night and temperatures of 24 °C/18 °C.

### 2.2. Microscopy

Anthers were immersed in formalin-aceto-alcohol (FAA) solution (Coolaber, Beijing, China) overnight. The dehydrated anthers were paraffin-embedded and sectioned at 8 μm using a microtome (RM2255, Leica, Wetzlar, Germany). Sections were stained using 0.01% (*w*/*v*) toluidine blue, followed by neutral resin sealing. After drying, they were observed with a microscope (DM2500, Leica, Wetzlar, Germany). Paraffin sectioning was performed with slight modifications based on previous studies [26].

### 2.3. Transcriptome Sequencing

RNA was extracted from 12 samples via the Plant Total RNA Isolation Kit (Vazyme, Nanjing, China). The number of anthers examined per stage was 30. RNA quality and concentration were measured by a Nanodrop 2000 (Thermo Scientific, Waltham, MA, USA). Only samples with RNA Integrity Number ≥ 7.6 were applied to library preparation. Each sample containing over 1 μg RNA was delivered to Novogene Bioinformatics Technology Co., Ltd. (Beijing, China). for sequencing on the Illumina HiSeq platform [27]. Reads containing adapters or with low quality or poly N were eliminated. The obtained clean reads were de novo-assembled with Trinity v2.1.1 [28]. The functional annotation of unigenes was performed across seven mainstream databases [29].

### 2.4. Differentially Expressed Gene Analysis

The expression of unigenes was assessed and normalized to fragments per kb per million reads (FPKM). Differential expression analysis was conducted with DESeq2 v1.4.5, and *p*-values were adjusted to control the false discovery rate [30]. DEGs were screened under the criteria of |log2(FoldChange)| ≥ 1 and adjusted *p* < 0.05 [31]. PCA, hierarchical cluster analysis and Venn diagram creation were performed using R software v4.2.1. Heatmaps were plotted via TBtools v2.210 [32]. Plant TFDB (https://planttfdb.gao-lab.org/aboutus.php, accessed on 18 November 2024) was used to predict the transcription factors [33]. Kyoto Encyclopedia of Genes and Genomes (KEGG) (https://www.kegg.jp/, accessed on 7 November 2024) and Gene Ontology (GO) (https://geneontology.org/, accessed on 5 November 2024) enrichment analyses were implemented in KOBAS 2.1.1 with *p*-adjust < 0.05.

### 2.5. Weight Gene Co-Expression Network Analysis (WGCNA)

WGCNA implemented in WGCNA R package (version 3.5.0) was used to explore the correlation between anther developmental stages and candidate genes based on the method described previously [34]. Parameters for WGCNA network construction were set as unsigned weighted network and dynamic hybrid tree-cut algorithm, combined with hierarchical clustering tree analysis, and minModuleSize of 30. A soft threshold of 12 (R^2^ > 0.9) was determined via the ‘pickSoftThreshold’ function to satisfy the scale-free topology criterion. Co-expression modules were detected by the ‘blockwiseModules’ function under the default parameters.

### 2.6. The qPCR Analysis

The cDNA was synthesized by a HiScript III 1st Strand cDNA Synthesis Kit (+gDNA wiper) (Vazyme, China). qPCR was carried out on StepOnePlus™ Real-Time PCR System (Applied Biosystems, Waltham, MA, USA) with ChamQ SYBR qPCR Master Mix (Vazyme, China) [27]. The reactions were established as follows: 8.2 µL of ultrapure water, 10 µL of SYBR, 1 µL of cDNA as template, and 0.35 µL of each primer. The conditions were 95 °C for 30 s, followed by 40 cycles of 95 °C for 10 s and 60 °C for 30 s. PCR specificity was confirmed by melting curve analysis. All experiments were conducted with three independent biological replicates. The relative expressions were determined using 2^−ΔΔCT^, with 18s rRNA and *Atactin* as reference genes for normalization [35,36]. Primers used for qPCR analysis were designed with Primer 5.0 (Table S1).

### 2.7. Subcellular Localization of PnMYB26

The CDSs of *PnMYB26* were amplified using the primers PnMYB26-S and PnMYB26-A (Table S1), and then ligated into pCAMBIA1302 (35S:GFP) vector to generate a reconstructed vector (35S:PnMYB26-GFP) [37]. 35S:PnMYB26-GFP together with 35S:GFP (control) was introduced into *Agrobacterium* (GV3101) (Vazyme, Nanjing, China), and then infiltrated into the leaves of tobacco [38]. After two days, infiltrated leaves were collected. DAPI was utilized for nucleus specific dye. Leaf tissues were incubated in 1 µg/mL DAPI (Thermo Scientific, Waltham, MA, USA) staining solution at room temperature for 10 min, with samples fully submerged in the staining liquid. Excess dye was washed off with sterile phosphate-buffered saline (PBS) (Solarbio, Beijing, China) three times before observation. The fluorescence signals were photographed with confocal microscopy (TCS SP2, Leica, Wetzlar, Germany).

### 2.8. Transgene Analysis of PnMYB26

Transgenic *Arabidopsis* plants were generated by transforming with 35S:PnMYB26-GFP and 35S:GFP via the floral dip method [39]. T0 seeds were collected and selected on MS medium (Solarbio, Beijing, China) containing 35 mg/L hygromycin (Solarbio, Beijing, China). Hygromycin-resistant seedlings were subsequently validated by PCR with PnMYB26-S and PnMYB26-A primers. Two transgenic lines (#6 and #7) were chosen for phenotype verification. Flowering and bolting time were calculated as the number of days after sowing. Each measurement was performed on 18 individual plants. Images of the flowers at anthesis were captured with a DM2500 microscope (Leica, Wetzlar, Germany). Floral buds were harvested prior to anthesis for qPCR analysis and pollen viability assay. Pollen viability was assessed via the Alexander staining method according to published protocols [40].

### 2.9. Statistical Analysis

Graphical representations were created with GraphPad Prism 8.0. All data were presented as the mean ± standard deviation (SD) from biological replicates. Comparisons between groups were made using Student’s *t*-test, with significance levels defined as *p* < 0.05 (*) and *p* < 0.01 (**). ns represents no significant difference.

## 3. Results

### 3.1. Transcriptome Assembly and Annotation

To dissect the molecular basis of anther development in *P. nigra*, anthers at four stages were collected for transcriptome sequencing (Figure S1A). Principal Component Analysis (PCA) showed that the samples were clearly separated, and replicates from the same developmental stage clustered together (Figure S1B). The correlation coefficient among biological replicates exceeded 0.8 (Figure S1C). These results showed that high-quality sequencing data were generated. In total, 264,894,824 clean reads were filtered from 272,550,109 raw reads (Table S2). After quality filtering, we generated 79.45 Gb clean data, corresponding to an average of 6.62 Gb per library. The GC content was between 50.34% and 53.57% in each sample, and the mapped reads ranged from 64.25% to 67.74%. All of the Q20 and Q30 values exceeded 98.87% and 96.76%, respectively, representing high quality sequencing. The Trinity assembly generated 124,735 unigenes with an N50 length of 1584 nt and a mean length of 1060 nt (Table S2). The longest and shortest unigenes were 17,710 and 301 bp, respectively. There were 43,747 (35.07%) unigenes of 200–500 bp in length, 39,585 (31.74%) unigenes ranged from 500 to 1000 bp, 24,126 (19.34%) unigenes had a length of 1000–2000 bp, and 17,277 (13.85%) unigenes were greater than 2000 bp.

The unigenes were aligned against seven public databases (NR, NT, PFAM, KO, KOG, SwissProt, and GO), and all 124,735 unigenes were annotated (Table S4). More specifically, the NR database annotated 64,621 (51.80%), the NT database annotated 75,558 (60.57%), the KO database annotated 20,844 (16.74%), the SwissProt database annotated 40,060 (32.11%), the PFAM and GO databases annotated 39,842 (31.94%), and the KOG database annotated 10,337 (8.28%) unigenes. In total, 90,976 unigenes (72.93%) obtained functional annotations in at least one database.

### 3.2. Differential Expression Analysis

**Figure 1:**
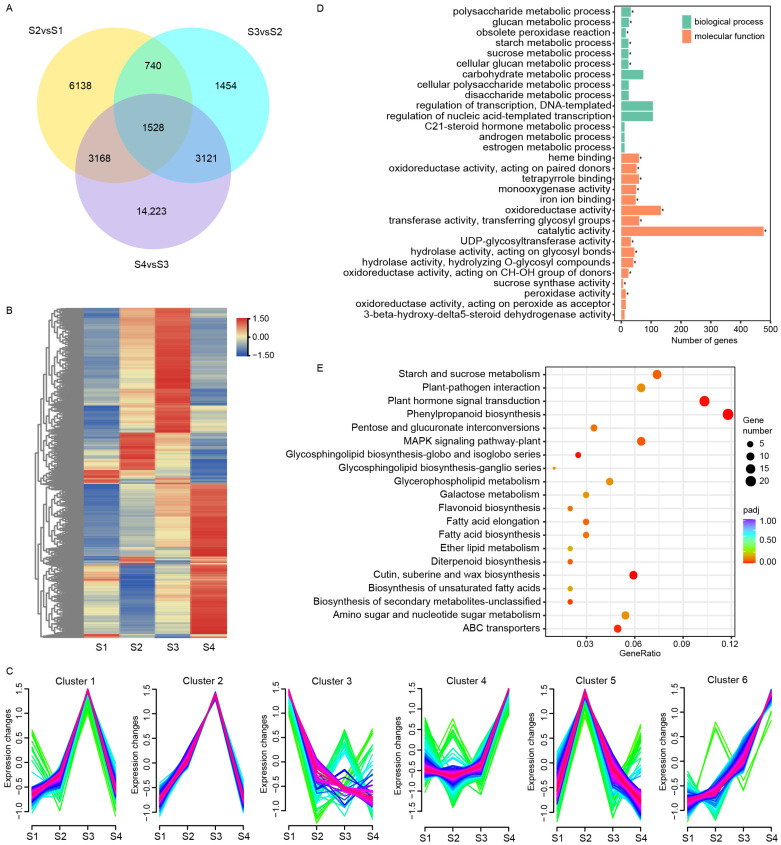
DEGs associated with stamen development. (**A**) Venn diagram of DEG numbers in the three comparative groups. (**B**) Expression profiles of the DEGs. (**C**) The six clusters with different gene expression pattern types. (**D**) GO functional analysis of the DEGs. * indicates that the corresponding term was significantly enriched (*p*-adjust < 0.05). (**E**) The 20 most significantly enriched KEGG pathways for the DEGs.

A total of 11,574 (6420 up and 5154 down), 6843 (5691 up and 1152 down), and 22,040 (11,281 up and 10,759 down) DEGs were identified in three comparisons (Figure S1D). The markedly higher number of DEGs detected in S4 relative to S3 underscores the unique gene expression pattern at this stage. In the comparison between S2 and S1, the top four most significantly enriched pathways were cutin, suberine and wax biosynthesis; plant hormone signal transduction; fatty acid biosynthesis; and phenylpropanoid biosynthesis. For the S3 vs. S2 comparison, ribosome was the primary significantly enriched pathway, with phenylpropanoid biosynthesis ranking second. For the S4 vs. S3 comparison, phenylpropanoid biosynthesis, fatty acid biosynthesis, and pentose and glucuronate interconversions were the top significantly enriched pathways (Figure S1E). These findings suggest that the phenylpropanoid biosynthesis pathway may function as a key regulator during *P. nigra* anther development. Finally, 1528 DEGs were detected in all three comparisons (Figure 1A). These DEGs were categorized into four groups (Figure 1B,C): 74 (cluster 3), 201 (cluster 5), 589 (cluster 1 and 2), and 664 (cluster 4 and 6) DEGs were specifically highly expressed at stages S1, S2, S3, and S4, respectively, implying that these DEGs (like *MYB26*, *MSP1* and *PTC1*) may function at distinct stages of anther development in *P. nigra*. Furthermore, more extensive gene changes were detected in the last two stages, suggesting that these phases are particularly critical for anther development.

### 3.3. GO Functional Annotation and KEGG Pathway Enrichment

An enrichment analysis of Gene Ontology (GO) terms was carried out, and the top 30 significantly enriched terms are presented in Figure 1D. The DEGs were categorized into molecular function and biological process. In the molecular function category, the DEGs were related to ‘heme binding’ and ‘oxidoreductase activity’. Meanwhile, the biological process category showed the strongest enrichment for ‘polysaccharide metabolic process’ and ‘glucan metabolic process’. These results demonstrated that anther development relied on multiple metabolic pathways, identifying the DEGs associated with these processes as key targets for future investigation.

To further characterize the functional pathways associated with the DEGs, we conducted KEGG pathway analysis (Figure 1E). Phenylpropanoid biosynthesis; cutin, suberine and wax biosynthesis; glycosphingolipid biosynthesis-globo and isoglobo series; ABC transporters; and plant hormone signal transduction were the top five pathways that may be associated with anther development. A total of twelve, twenty-four, and ten DEGs were associated with cutin, suberine and wax biosynthesis, phenylpropanoid biosynthesis, and ABC transporters, respectively. These DEGs (like *FAR2-1*, *FAR2-2*, *CYP704B1*, *4CL*, *CAD6*, *CCR1*, and *ABCG26*) may be involved in anther development.

### 3.4. WGCNA

**Figure 2:**
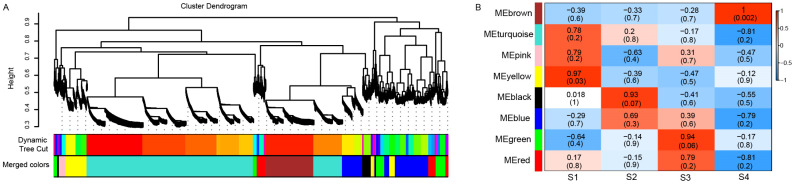
WGCNA of anther development in *P. nigra*. (**A**) Hierarchical clustering dendrogram displaying the identified co-expression modules. (**B**) Module–sample association analysis.

WGCNA identified eight co-expression modules from 35,003 genes across the four developmental stages (Figure 2A and Table S5). The correlation analysis showed that four modules exhibited correlations with the distinct stages: MEyellow (r = 0.97, *p* = 0.003), MEblack (r = 0.93, *p* = 0.07), MEgreen (r = 0.94, *p* = 0.06), and MEbrown (r = 1, *p* = 0.002) (Figure 2B). To visualize the interactions within these key modules, gene co-expression networks were constructed (Figure S2A). A total of 264 common genes were identified between these four modules and the DEGs (Figure S2B). KEGG enrichment analysis showed that these 264 common genes were primarily associated with six key pathways, including flavonoid biosynthesis; cutin, suberine and wax biosynthesis; plant hormone signal transduction; and phenylpropanoid biosynthesis (Figure S2C). Notably, these genes function in cutin, suberine and wax biosynthesis as well as phenylpropanoid biosynthesis, and may serve critical roles in anther development.

### 3.5. Differentially Expressed Transcription Factors

**Figure 3:**
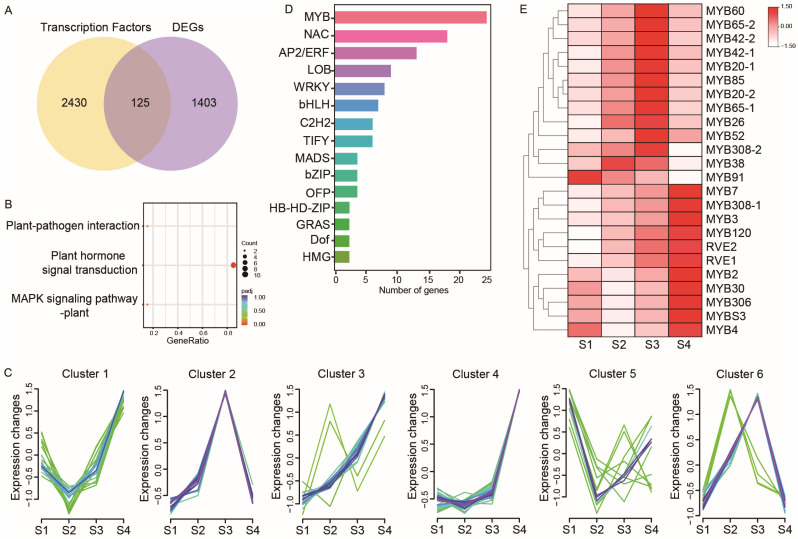
Differentially expressed transcription factor analysis of stamen development. (**A**) Venn diagram of the numbers between the DEGs and the transcription factors. (**B**) KEGG enrichment analysis. (**C**) The six clusters of different expressed transcription factors with different expression pattern types. (**D**) Major families represented among the differentially expressed transcription factors. (**E**) Heatmap of MYB transcription factors.

We identified 125 transcription factors encoded by the 1528 common DEGs, which belonged to 26 different families (Figure 3A). KEGG enrichment analysis demonstrated that these transcription factors were most significantly associated with the plant hormone signal transduction pathway (Figure 3B). Cluster analysis classified 125 transcription factors into six clusters (Figure 3C). The transcription factors in cluster 5 were predominantly expressed in S1, those in cluster 2 peaked at S3, and those in clusters 1, 3, and 4 showed the highest expression in S4. Meanwhile, cluster 6 maintained relatively high expression levels in S2 and S3. The largest transcription factor family was MYB (Figure 3D), which precisely regulated flower development by integrating environmental stimuli and hormonal signals [41]. The heatmap of 24 MYB genes displayed distinct expression patterns across developmental stages: one MYB showed high expression at S1, one at S2, eleven at S3, and eleven at S4 (Figure 3E). This suggests that these MYBs function predominantly during the uninucleate microspore and binucleate pollen stages. Among these MYBs, several showing significant differential expression were identified as key regulators, such as *MYB26*, *MYB65-1* and *MYB65-2*, in which *MYB26* was also identified within the MEgreen module of WGCNA.

### 3.6. qPCR Validation

**Figure 4:**
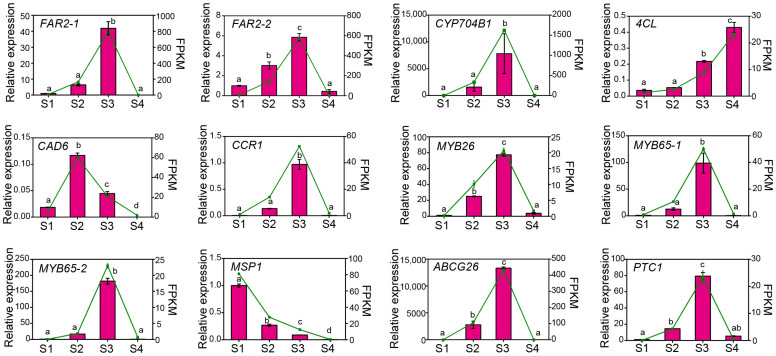
Validation of 12 DEGs by qPCR. The bars stand for the relative expression of the qPCR result and the lines represent the FPKM value of the RNA-seq result. All qPCR data are presented as mean ± SD. One-way ANOVA was used to test intergroup differences, with different lowercase letters indicating statistically significant differences (*p* < 0.05).

Twelve genes with high fold change and critical roles in anther development were selected for qPCR (Figure 4). These genes were mainly chosen from DEGs involved in cutin, suberine and wax biosynthesis (*FAR2-1*, *FAR2-2*, and *CYP704B1*), phenylpropanoid biosynthesis (*4CL*, *CAD6* and *CCR1*), and ABC transporters (*ABCG26*). In addition, three transcription factors (*MYB26*, *MYB65-1* and *MYB65-2*) and two DEGs (*MSP1* and *PTC1*) with essential functions were also selected. A comparison of qPCR and transcriptome data showed strong concordance in expression patterns, confirming the accuracy and reliability of our transcriptomic analysis.

### 3.7. Subcellular Localization of PnMYB26

**Figure 5:**
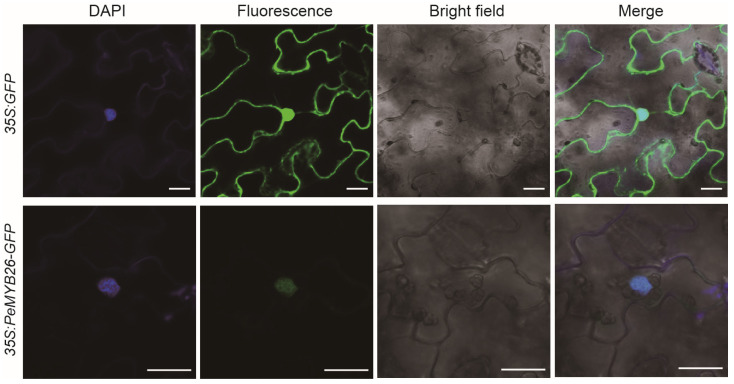
Subcellular localization of PeMYB26 in tobacco epidermal cells. The first row presents DAPI staining, GFP fluorescence, bright field and merged photographs for the 35S:GFP; the second row shows the corresponding four images for the 35S:PnMYB26-GFP fusion protein. Empty GFP is distributed across the cytoplasm and nucleus, whereas PnMYB26-GFP signals are co-localized with nuclear DAPI staining, demonstrating the nuclear localization of PnMYB26. Scale bar = 20 µm.

To investigate the subcellular localization of PnMYB26, we transiently expressed 35S:PnMYB26-GFP and 35S:GFP. Confocal microscopy analysis revealed that PnMYB26 was predominantly localized in the nucleus, colocalized with the nuclear marker. In contrast, control was localized in both the cytoplasm and nucleus (Figure 5). This indicated that PnMYB26 may be involved in cellular function.

### 3.8. Overexpression of PnMYB26 Led to Markedly Reduced Fertility

To further explore the function of *PnMYB26*, we overexpressed *PnMYB26* in *Arabidopsis*. Eight transgenic lines were generated and verified by PCR (Figure S3). Among these lines, two homozygous lines (#6 and #7) were selected. No significant differences in bolting or flowering time were observed between PnMYB26-OE and the control plants (Figure S4), indicating that *PnMYB26* had no effect on flowering.

**Figure 6:**
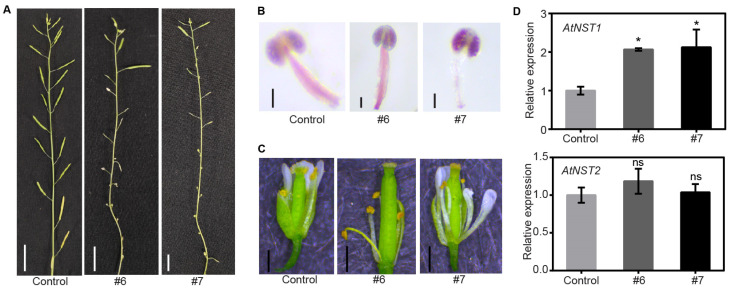
Phenotypes of transgenic plants overexpressing *PnMYB26*. (**A**) Phenotypes of siliques in the transgenic plants. Scale bar = 1 cm. (**B**) Alexander staining for pollen viability in transgenic plant anthers. Scale bar = 100 µm. (**C**) Flower morphology of transgenic plants. Scale bar = 500 µm. (**D**) Transcript levels of *NST1* and *NST2* in the transgenic plants. Statistical significance is determined using Student’s *t*-test. Asterisks indicate significant differences: * *p* < 0.05; ns denote no statistically significant difference.

Compared with the control, PnMYB26-OE plants exhibited a significantly reduced seed-setting rate, and most of their pods were abnormal with no seed formation (Figure 6A). No significant difference in pollen viability was observed between the transgenic lines and the control (Figure 6B). Interestingly, abundant pollen from control plants was obviously dispersed on the stigmas, while anthers of transgenic lines positioned below the pistil and no pollen grains were present on stigmas of the overexpression plants (Figure 6C). These results revealed that the overexpression of *PnMYB26* impaired anther dehiscence, which ultimately led to markedly reduced fertility.

In *Arabidopsis*, *MYB26* regulates anther secondary wall thickening by promoting *NST*s [20]. Consistent with this, *NST1* was significantly elevated in transgenic plants (Figure 6D), suggesting that the overexpression of *PnMYB26* modulated the expression of *NST1*. We also calculated pairwise expression correlation coefficients across all anther developmental samples and constructed a targeted expression correlation network (Figure S5). This network demonstrated that *PnMYB26* closely correlated with numerous transcription factors, including MYB, LBD and NAC, which play roles in anther development or secondary wall formation. Collectively, our findings demonstrate that the overexpression of *PnMYB26* may drive abnormal secondary wall thickening in the anther endothecium, ultimately resulting in male gametophytic abortion.

## 4. Discussion

For plant sexual reproduction, anther development is a critical and multi-layered process governed by a series of regulatory genes [6]. In this study, we systematically investigated the transcriptomic dynamics of four anther development processes of *P. nigra* by sequencing. A total of 1528 common DEGs related to anther development were identified (Figure 1A). These genes were mainly enriched in phenylpropanoid biosynthesis and in cutin, suberine and wax biosynthesis (Figure 1E), consistent with previous studies in maize [42]. Such consistent pathway enrichment indicates that these two metabolic processes are core and conserved modules governing plant anther development. Phenylpropanoid metabolism provides precursors for lignin, essential for endothecial secondary wall thickening. Through differential expression analysis, WGCNA, KEGG analysis, transcription factor analysis and qPCR, twelve genes were considered key genes related to anther development (Figure 4). Furthermore, the ectopic overexpression of *PnMYB26* led to male gametophyte abortion and a decline in seed-setting rate (Figure 6). This study offers preliminary molecular insights into anther development in *P. nigra*.

The anther cuticle, primarily made up of cutin and wax, protects pollen against external environmental conditions [43]. Numerous genes regulating anther cuticle and exine formation have been uncovered, such as *FAR2* and *PnCYP704B1*. *AtFAR2* contributes to the synthesis of key components necessary for pollen exine formation. Loss-of-function mutations in *AtFAR2* lead to defective pollen wall development [8]. *CYP704B1* mediates the co-hydroxylation of long-chain fatty acids, which is essential for sporopollenin synthesis. Mutations in *AtCYP704B1* lead to a lack of normal exine layer and cause defective pollen walls [9]. *PnFAR2-1*, *PnFAR2-2*, and *PnCYP704B1* were also identified as being highly expressed in stage S3 (Figure 4). The stage-specific upregulation of these genes demonstrates the robust activation of cuticle and pollen wall biosynthesis during the middle anther developmental stage in *P. nigra*. Their conserved expression profiles also reveal the functional conservation of pollen wall regulatory pathways across *P. nigra* and model plants.

Phenylpropanoid metabolism generates numerous important secondary metabolites, such as flavonoids and lignin, which are essential for plant anther development [10]. Endothecial lignification and cell wall thickening govern anther dehiscence and further affect fertility [44]. In phenylpropanoid biosynthesis metabolism, two critical enzymes catalyze the conversion of phenylalanine to cinnamoyl-CoA: 4-coumarate-CoA ligase and phenylalanine ammonia lyase [45]. Cinnamoyl-CoA is transformed into flavonoids via chalcone synthase, supporting normal pollen development and maturation [46]. Lignin monomer synthesis, required for cell wall formation, is controlled by cinnamyl alcohol dehydrogenase (CAD) and cinnamoyl-CoA reductase (CCR) [47]. The mutant of *Gh4CL20* shows indehiscent anthers and leads to male sterility [11]. The phenotypic analysis of cotton knockout and overexpression lines demonstrated that *GhCAD37* governs lignin biosynthesis within anther vascular bundles, thereby regulating anther viability [48]. The *ccr1 cadc cadd* triple mutant of *Arabidopsis* exhibits severe male sterility in *Arabidopsis*. The underlying cause is inadequate lignification within the anther endothecium, which further leads to defective anther dehiscence and impaired pollen dispersal [12]. In this study, *PnCAD6*, *PnCCR1* and *Pn4CL* were identified and highly expressed in stage S2, S3, and S4, respectively (Figure 4). The differential temporal expression reveals tight temporal control of lignin biosynthesis in developing *P. nigra* anthers, with enzymatic genes functioning sequentially to ensure orderly cell wall thickening and anther maturation.

Moreover, 125 transcription factors related to anther development were identified (Figure 3A). Among all transcription factor families, the MYB family was the most abundant (Figure 3D). MYBs play important roles in male reproductive processes, including anther dehiscence and pollen development [49]. The overexpression of *AtMYB26* in tobacco upregulates *NST1* expression, triggering abnormal thickening of the anther wall layers and impairing anther dehiscence, ultimately leading to complete male sterility [41]. Here, PnMYB26-overexpressing plants also exhibited decreased male fertility, which was attributed to impaired anther dehiscence (Figure 6A,C). Furthermore, *NST1* was upregulated in PnMYB26-overexpressing plants (Figure 6D). In *P. nigra*, *PnMYB26* declined significantly at S4 stage, a stage associated with endothecial secondary wall thickening and anther dehiscence. Reduced *PnMYB26* expression may hinder the activation of downstream *NST1*, resulting in defective secondary wall thickening. This ultimately causes incomplete or delayed anther dehiscence and low natural seed set.

The JAZ-PRC repressive complex negatively regulates *AtMYB26* to modulate stamen development, so that suppresses excessive JA signaling in male reproductive processes [50]. Interestingly, KEGG enrichment analysis revealed that 125 transcription factors may be involved in hormone signaling (like JA) (Figure 3B). These findings indicate that the PnMYB26-*NST1* regulatory module, together with JA-mediated hormone signaling, may participate in the control of *P. nigra* anther development and male fertility. Further investigations are necessary to determine the specific mechanism mediated by *PnMYB26* in controlling anther development in *P. nigra*.

## 5. Conclusions

Anther development is a highly complex process mediated by multiple regulatory genes. Here, 1528 DEGs related to anther development were detected by transcriptome sequencing. KEGG analysis showed that these DEGs were predominantly enriched in the cutin, suberine and wax biosynthesis and phenylpropanoid biosynthesis pathways. Combined with WGCNA and gene profiling, 12 genes closely related to anther development were screened. Moreover, *PnMYB26* was shown to regulate fertility by controlling anther dehiscence. Collectively, these results provide a solid theoretical foundation for future studies on anther development in bamboo.

## Data Availability

The original contributions presented in this study are included in the article/Supplementary Materials. Further inquiries can be directed to the corresponding authors.

## Supplementary Materials

The following supporting information can be downloaded at https://www.mdpi.com/article/10.3390/biology15131049/s1: Figure S1. (A) Cytological observation of anthers from *P. nigra*. Scale bar = 50 µm. (B) Correlation analysis heatmap of *P. nigra* anther at the four stages. Color intensity indicates the correlation level among samples. (C) PCA for the transcriptome of anthers under the four stages. (D) DEG analysis of the three comparison groups. (E) KEGG enrichment results for DEGs. Figure S2. (A) Visualization of hub gene networks from the four WGCNA modules. Each node represents a gene, and edges represent gene–gene connections. Nodes with a darker color indicate higher connectivity. Genes without functional annotation are not labeled. (B) Venn diagram of the numbers between the four modules and DEGs. (C) KEGG pathway enrichment of the 264 DEGs. Figure S3. RT-PCR validation of the transgenic plants overexpressing *PnMYB26*. Figure S4. Observation of flowering in the transgenic plants overexpressing *PnMYB26*. (A) Flowering phenotype in the transgenic plants. Scale bar = 1.5 cm. Days of bolting (B) and flowering (C) in the transgenic plants. Figure S5. Expression correlation network of *PnMYB26*. The central node is PnMYB26, connected to a set of anther development-related transcription factors with high expression correlation. Table S1. Primers used in this study. Table S2. Overview of transcriptome sequencing data. Table S3. Transcriptome unigene results. Table S4. Annotation details of the assembled unigenes. Table S5. The genes in different modules.
