# Supplementary figures and images for: Transcriptome Analysis Reveals the Involvement of PnMYB26 in Regulating Anther Development in Phyllostachys nigra
Source: Biology (Basel). 2026 Jul 1;15(13):1049. doi: 10.3390/biology15131049 (PMC13360393; doi:10.3390/biology15131049)

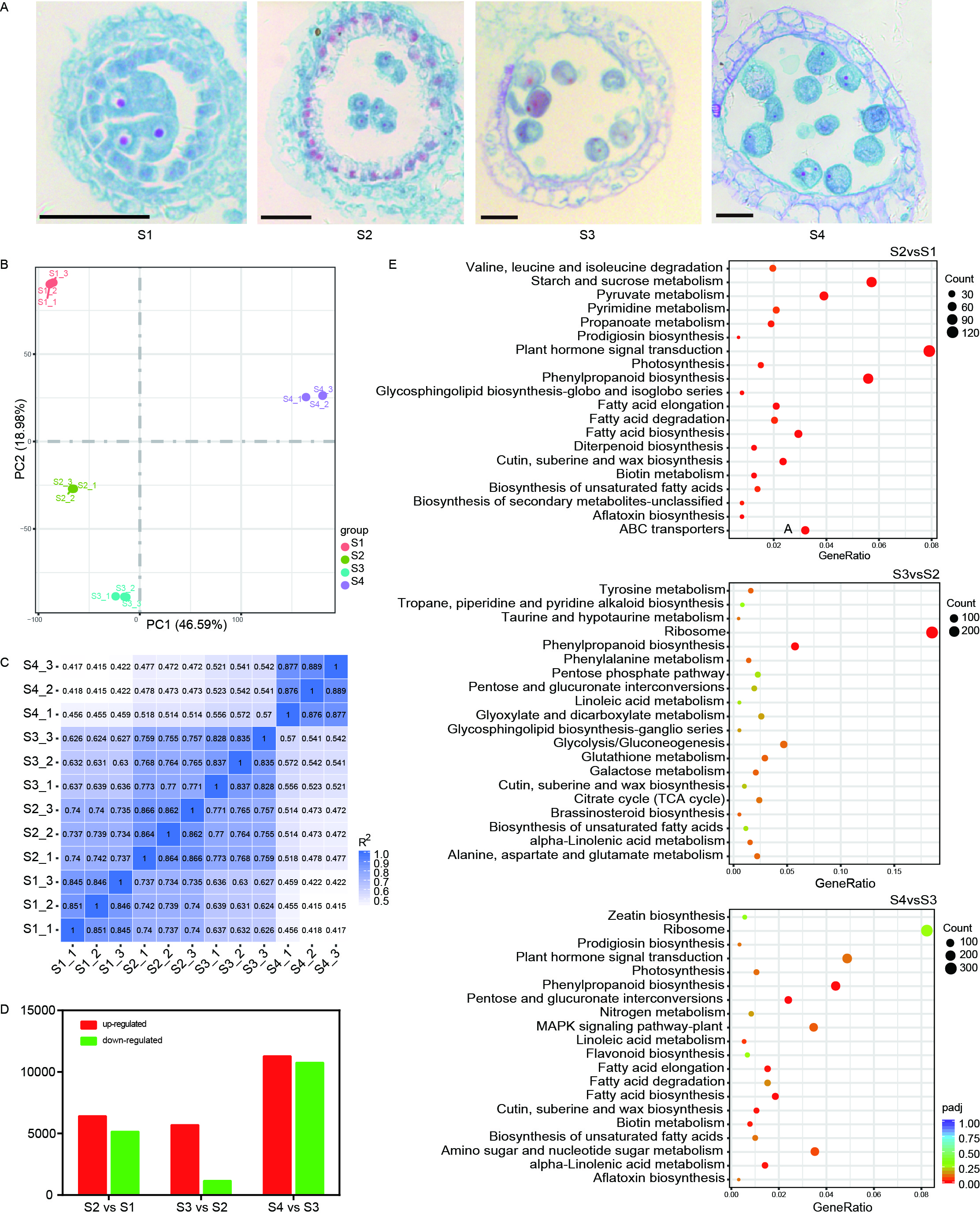

Supplement: Supplementary file 1 [file biology-15-01049-s001.zip › Fig. S1.jpg]

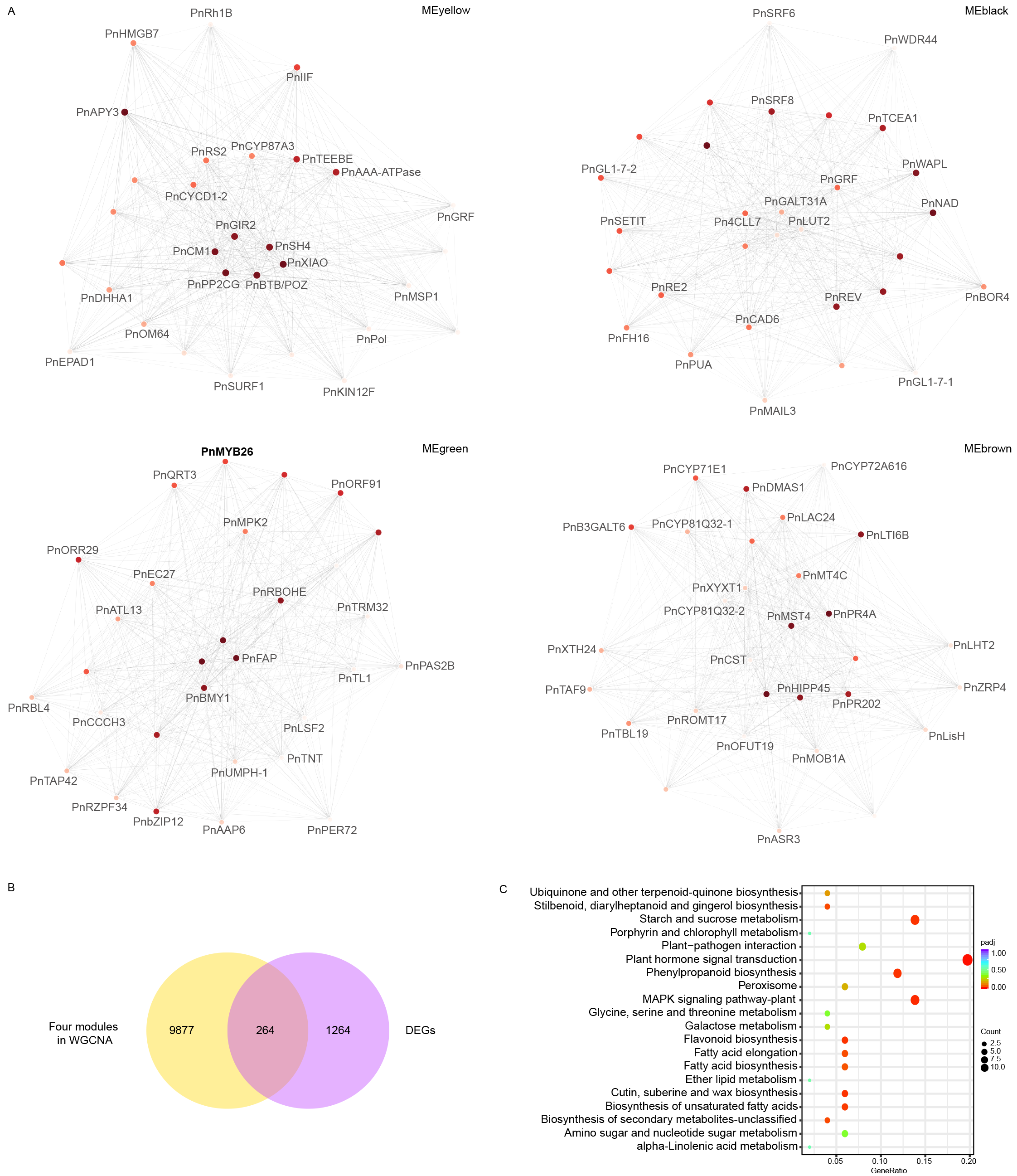

Supplement: Supplementary file 1 [file biology-15-01049-s001.zip › Fig. S2--2026.6.26.tif]

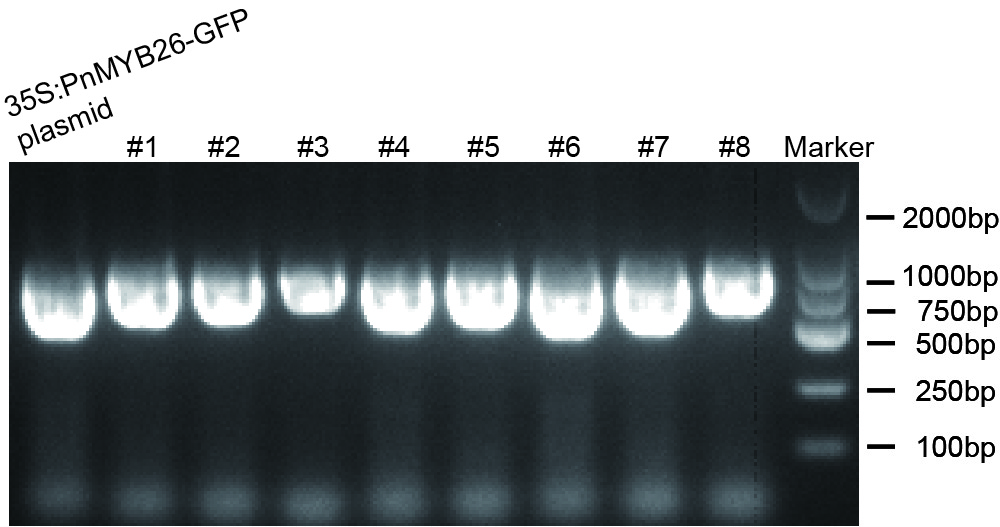

Supplement: Supplementary file 1 [file biology-15-01049-s001.zip › Fig. S3--2026.6.10.jpg]

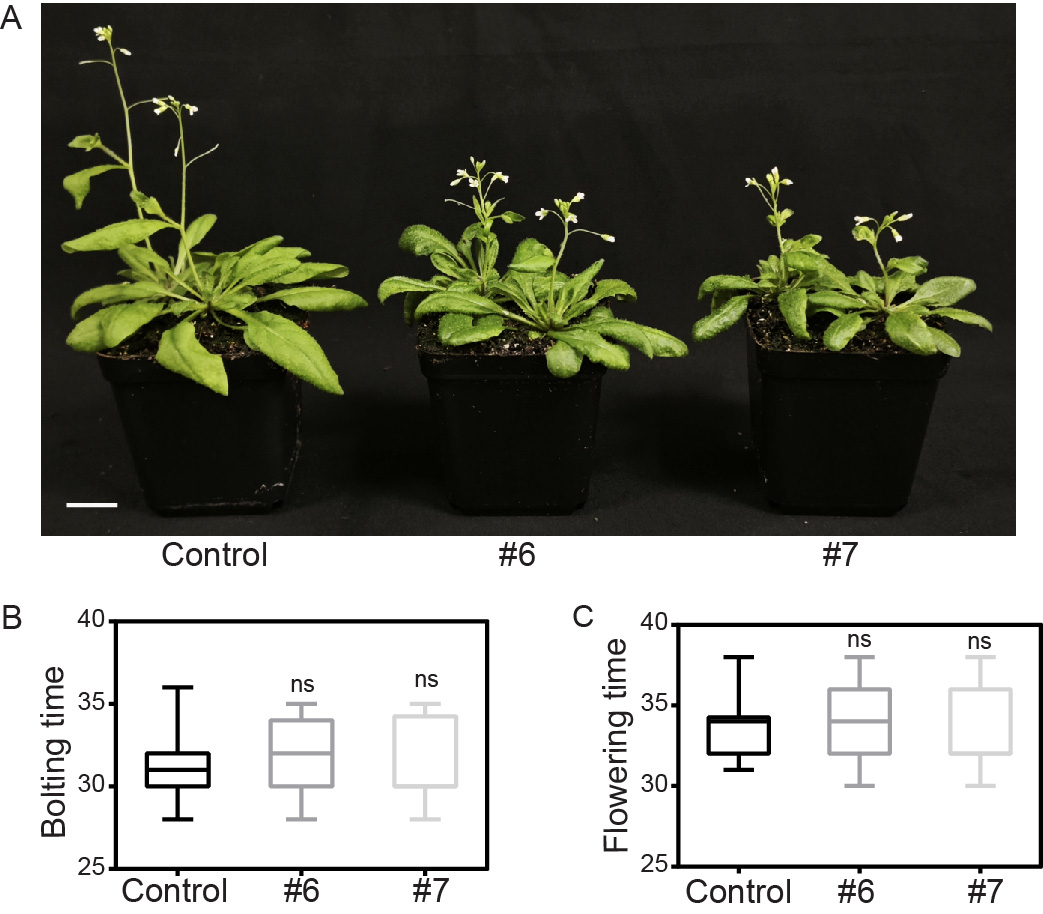

Supplement: Supplementary file 1 [file biology-15-01049-s001.zip › Fig. S4-标尺1.5cm.jpg]

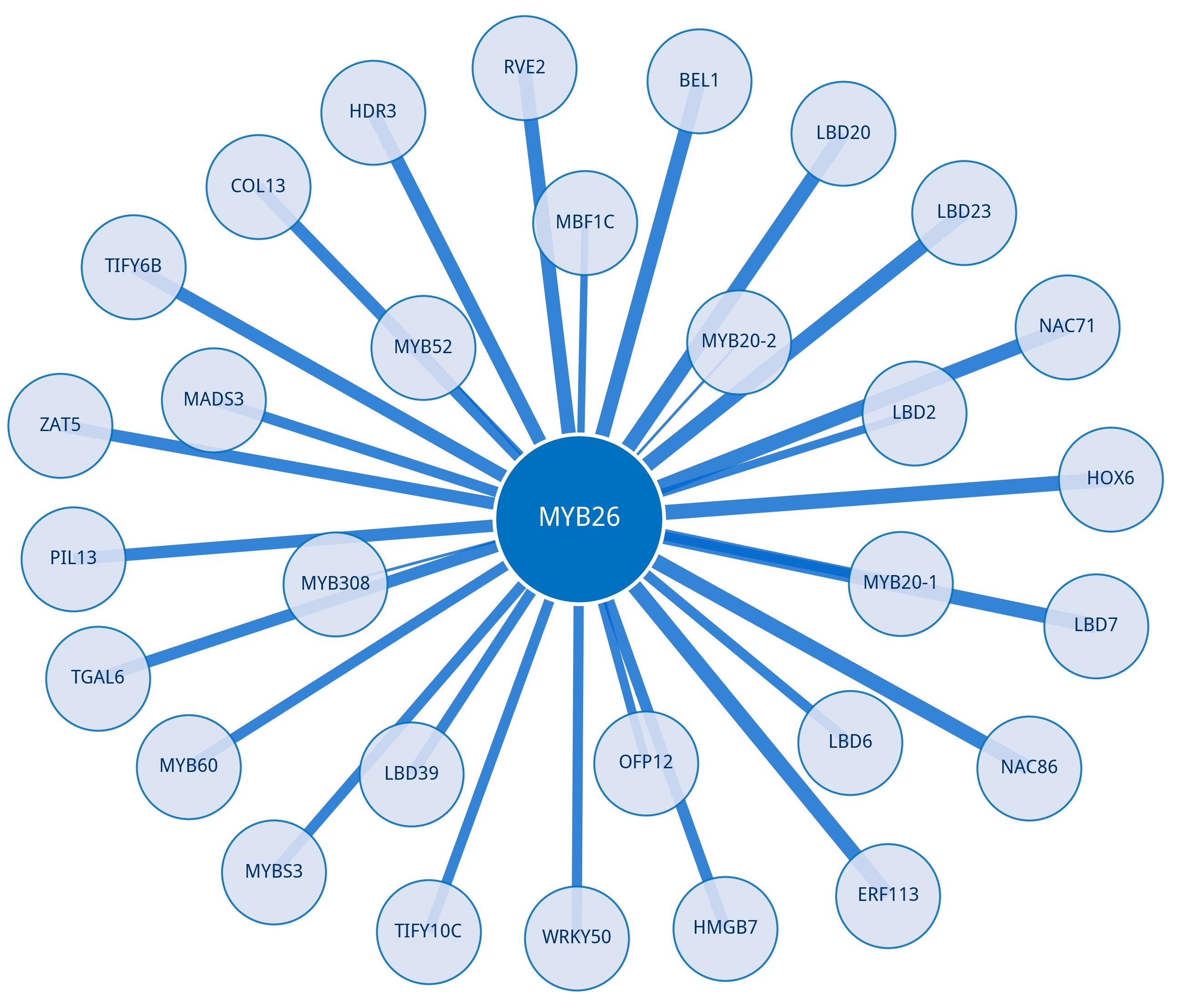

Supplement: Supplementary file 1 [file biology-15-01049-s001.zip › Fig. S5--2026.6.16.jpeg]
